# Simulation-based team training for healthcare professionals in pediatric departments: study protocol for a nonrandomized controlled trial

**DOI:** 10.1186/s12909-024-05602-z

**Published:** 2024-06-01

**Authors:** A. Schram, N. L. Bonne, T. B. Henriksen, C. Paltved, N. T. Hertel, M. S. Lindhard

**Affiliations:** 1 Corporate HR, MidtSim, Central Denmark Region, Aarhus, Denmark; 2 Department of Paediatrics and Adolescent Medicine, Aarhus University Hospital, Aarhus, Denmark; 3 Department of Clinical Medicine, Aarhus University, Aarhus, Denmark; 4 HC Andersen Childrens Hospital, Odense University Hospital, Odense, Denmark; 5 Department of Pediatrics, Regional Hospital of Randers, Randers, Denmark

**Keywords:** Simulation-based team training, Pediatrics, Complex intervention

## Abstract

**Background:**

Healthcare systems worldwide face challenges related to patient safety, quality of care, and interprofessional collaboration. Simulation-based team training has emerged as a promising approach to address some of these challenges by providing healthcare professionals with a controlled and safe environment to enhance their teamwork and communication skills. The purpose of this study protocol is to describe an intervention using simulation-based team training in pediatric departments.

**Methods:**

Using a parallel-group, non-randomized controlled trial design, a simulation-based team training intervention will be implemented across four pediatric departments in Denmark. Another four pediatric departments will serve as controls. The intervention implies that healthcare professionals engage in simulation-based team training at a higher quantity and frequency than they did previously. Development of the intervention occurred from April 2022 to April 2023. Implementation of the intervention occurs from April 2023 to April 2024. Evaluation of the intervention is planned from April 2024 to April 2025. All simulation activity both before and during the intervention will be registered, making it possible to compare outcomes across time periods (before versus after) and across groups (intervention versus control). To evaluate the effects of the intervention, we will conduct four analyses. Analysis 1 investigates if simulation-based team training is related to sick leave among healthcare professionals. Analysis 2 explores if the simulation intervention has an impact on patient safety culture. Analysis 3 examines if simulation-based team training is associated with the treatment of critically ill newborns. Finally, Analysis 4 conducts a cost-benefit analysis, highlighting the potential return on investment.

**Discussion:**

The implemented simulation-based team training intervention can be defined as a complex intervention. Following the Medical Research Council framework and guidelines, the intervention in this project encompasses feasibility assessment, planning of intervention, implementation of intervention, and rigorous data analysis. Furthermore, the project emphasizes practical considerations such as stakeholder collaboration, facilitator training, and equipment management.

**Trial registration:**

Registered as a clinical trial on clinicaltrials.gov, with the identifier NCT06064045.

**Supplementary Information:**

The online version contains supplementary material available at 10.1186/s12909-024-05602-z.

## Background

Healthcare systems worldwide face numerous challenges that impact patient safety, quality of care, interprofessional collaboration, workplace psychological safety, and staff turnover [1, 2]. Factors such as communication breakdowns, medical errors, and lack of coordination among healthcare professionals, contribute to adverse patient outcomes, reduced job satisfaction, and inefficiencies within healthcare delivery [3, 4]. These challenges underscore the urgent need for innovative approaches that foster effective teamwork, enhance communication skills, and promote collaboration among healthcare professionals [5].

Simulation-based team training is a supportive initiative in healthcare [6]. This training methodology utilizes simulated scenarios that closely mirror real-life clinical situations, providing healthcare professionals with a controlled environment to practice and enhance their teamwork skills [7]. It involves multidisciplinary teams working together to solve problems, make decisions, and communicate effectively, thereby improving their collective performance [8]. The use of simulation-based team training in healthcare holds several advantages. Firstly, it allows healthcare professionals to develop and refine their teamwork skills in a safe and risk-free environment [9]. Secondly, it provides an opportunity for interprofessional collaboration, enabling healthcare professionals from different disciplines to work together, understand each other’s roles, and foster a collaborative culture [10–12]. Thirdly, simulation-based team training promotes a safe culture of continuous learning and improvement, supporting the mindset that errors can and will happen, and that proactively learning from them will increase treatment quality and patient safety [13, 14]. It allows healthcare professionals to practice critical skills, such as effective communication, shared decision-making, and situational awareness, which are essential for delivering safe and coordinated care [15]. By supporting healthcare professionals, simulation-based team training ultimately aims to improve the quality of care. Nevertheless, there is a requirement for more comprehensive and inclusive research, focused on the utilization of simulation-based team training.

First, much of the existing literature exploring the impact of simulation predominantly features single or few training sessions of limited duration. On the contrary, there is a need for research that emphasizes extended exposure to simulation experiences [10, 16–18]. A more powerful exposure can potentially unlock a deeper level of skill acquisition, better team dynamics, and a more profound impact on real-world performance. Secondly, a knowledge gap exists in terms of studies including (1) a higher number of participants, (2) control groups, (3) measurements of long-term effects, and (4) cost-benefit analysis [19–25]. Finally, a more comprehensive and holistic approach can amalgamate insights from multiple disciplines, encompassing psychology, education, and human factors [26–28]. This interdisciplinary methodology provides a more thorough understanding of the impact of simulation-based team training. Given the substantial diversity in simulation teams in terms of individual attributes, team size, and objectives, there is a compelling need for research aimed at optimizing the utilization of team dynamics across various professions and personal backgrounds. Conducting this research is pivotal in tailoring initiatives to cater to the distinct needs of different teams [11, 12, 29].

To elucidate such a broad spectrum of factors and characteristics, a more complex intervention is required. Aligned with the identified research needs, the Medical Research Council framework posits that complex interventions leverage complexity as a tool to foster more holistic, effective, and sustainable healthcare outcomes [30].

Although the necessity for implementing a complex intervention involving simulation-based team training is evident, it comes with its unique array of implementation challenges. Healthcare organizations and professionals may encounter several obstacles when integrating or regulating new initiatives into their daily routines. Recognizing and addressing these challenges is crucial for successfully adopting and sustaining simulation-based team training programs. Some of the key challenges include; early involvement of stakeholders, resource allocation, time constraints, resistance to change among employees, sustainability, and a cultural shift [30–35].

Considering the multifaceted challenges at hand, this study protocol seeks to delineate the design, execution, and evaluation of a comprehensive intervention centered on simulation-based team training. The overarching perspective is to support healthcare professionals and thus, improve quality of care. To investigate the impact of a simulation-based team training intervention, we aim to conduct four analyses, including (1) sick leave among healthcare professionals, (2) patient safety culture in hospital departments, (3) quality of treatment of critically ill newborns, and lastly, (4) a cost-benefit analysis.

### Hypothesis

We anticipate that the simulation-based team training intervention may result in:

- Decreased sick leave rates.
- Enhanced patient safety culture.
- Improvement in Apgar scores between one and 10 min after birth.
- Cost-related benefits.

## Methods

The trial design in this project is defined as a parallel group, non-randomized controlled trial. The trial was registered at clinicaltrials.gov, with the identifier NCT06064045. The SPIRIT checklist and SPIRIT schematic diagram were used when writing this protocol, which can be found in Appendix 1 and Appendix 2 [36].

### Setting

This project will be implemented in four pediatric departments in Denmark. A simulation-based team training intervention will be implemented in one healthcare region in Denmark. Concurrently, another Danish healthcare region will serve as the control group, in which no intervention will be implemented. Both regions serve approximately 1,200,000 residents and operate one university hospital and three regional hospitals. Pediatric departments and neonatal intensive care units are located in all hospitals. Both healthcare regions include a total of approximately 600 healthcare professionals, leading to a total of approximately 1,200 staff participants.

### Intervention

From April 2023 to April 2024, a simulation-based training program will be implemented in the intervention region. All healthcare professionals (n = approx. 600) working as physicians or nurses in the pediatric departments in the intervention region will be eligible to participate. The intervention is defined as an educational program and includes;

#### Participants in the intervention group engage in simulation-based team training at a higher quantity and frequency

**Fig. 1:**
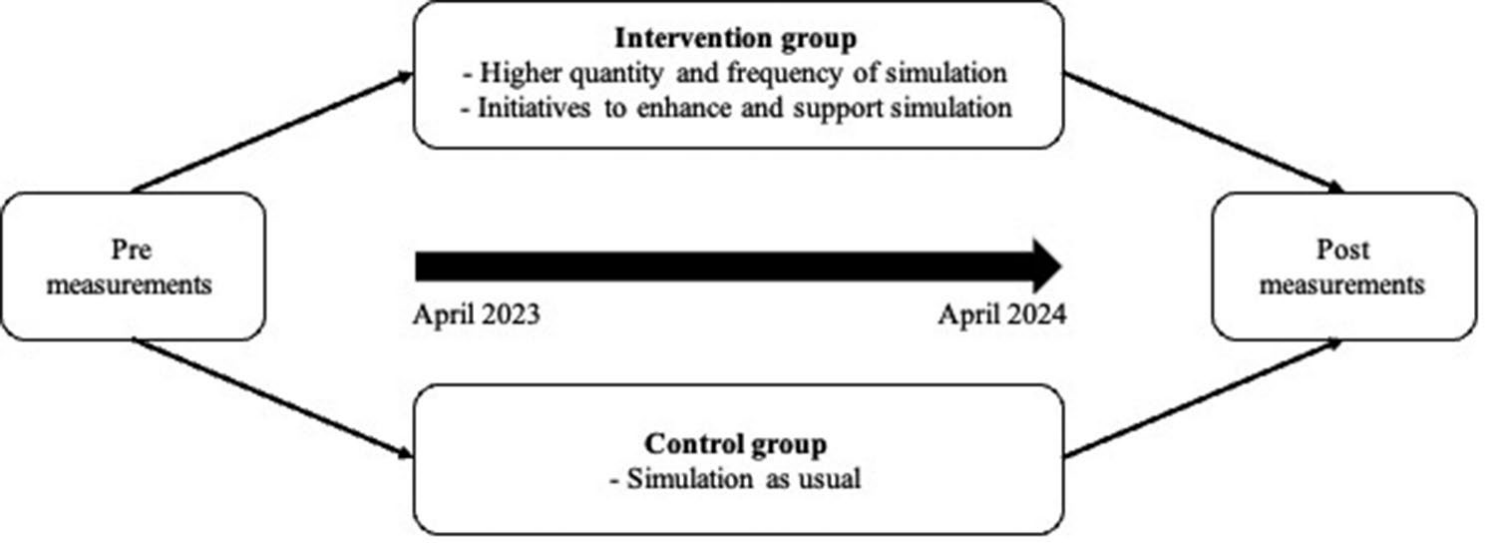
Process of intervention

This entails that healthcare professionals in the intervention region are (1) participating in more simulation and (2) engaging in simulation activities at a higher frequency than what was previously considered customary. During the daytime work hours, all simulation sessions will occur in the hospital departments (in situ). Simulation scenarios were developed by simulation experts within the field of pediatrics and include either newborns or children. Currently, simulation-based training is an integral part of daily working hours at hospitals in developing countries. To gain an understanding of the extent of simulation activities across all participating pediatric departments, we conducted a three-month registration process to document all facilitated simulations before the intervention. The intervention group facilitated a total of 27 simulation sessions, whereas the control group facilitated a total of 22 simulation sessions across these three months. Anticipated outcomes involve the control group maintaining their standard simulation practices, amounting to 88 sessions throughout the intervention period. In contrast, we expect the intervention group to participate in four times the number of simulation sessions, totaling 352, within the identical time frame. While this is an anticipation and objective, it is not a compulsory requirement within the individual departments. In addition to the amount and frequency of simulation, specific initiatives to enhance and support simulation will be implemented in the intervention region. Figure 1 illustrates the intervention process.

The project is planned to span three years, commencing in April 2022 and concluding in April 2025. The development phase of the project occurred from April 2022 to 2023, whereas the intervention was implemented from April 2023 to April 2024, and finally, the evaluation is planned from April 2024 to April 2025 (Appendix 1).

### Development and implementation of the intervention

To provide a clear and visual representation of the key components and relationships involved in the intervention, we applied a logic model during the development of the simulation-based team training intervention (see Table 1). The model functions as a strategic guide for planning and mapping out pathways by defining objectives, identifying inputs, outlining activities, and specifying both expected outputs and outcomes.

**Table 1:** Logic model visualization of simulation-based team training intervention

Objectives	Inputs	Activites	Output	Intervention outcomes	Distal outcomes (not directly measured)
To investigate the impact of a simulation-based team training intervention	- Collaboration with stakeholders - Preparing simulation facilitators - Acquisition of equipment - Registering all performed simulation	Participants engage in simulation-based team training at a higher quantity and frequency	Hours of performed simulation	- Sick leave - Patient safety culture - Apgar score - Cost benefit	- Improved patient safety - Increased job satisfaction

During the development of the simulation-based team training, the following inputs were prioritized:

- Collaboration with stakeholders.
- Preparing simulation facilitators.
- Acquisition of equipment.
- Registering all performed simulations.

#### Collaboration with stakeholders

**Fig. 2:**
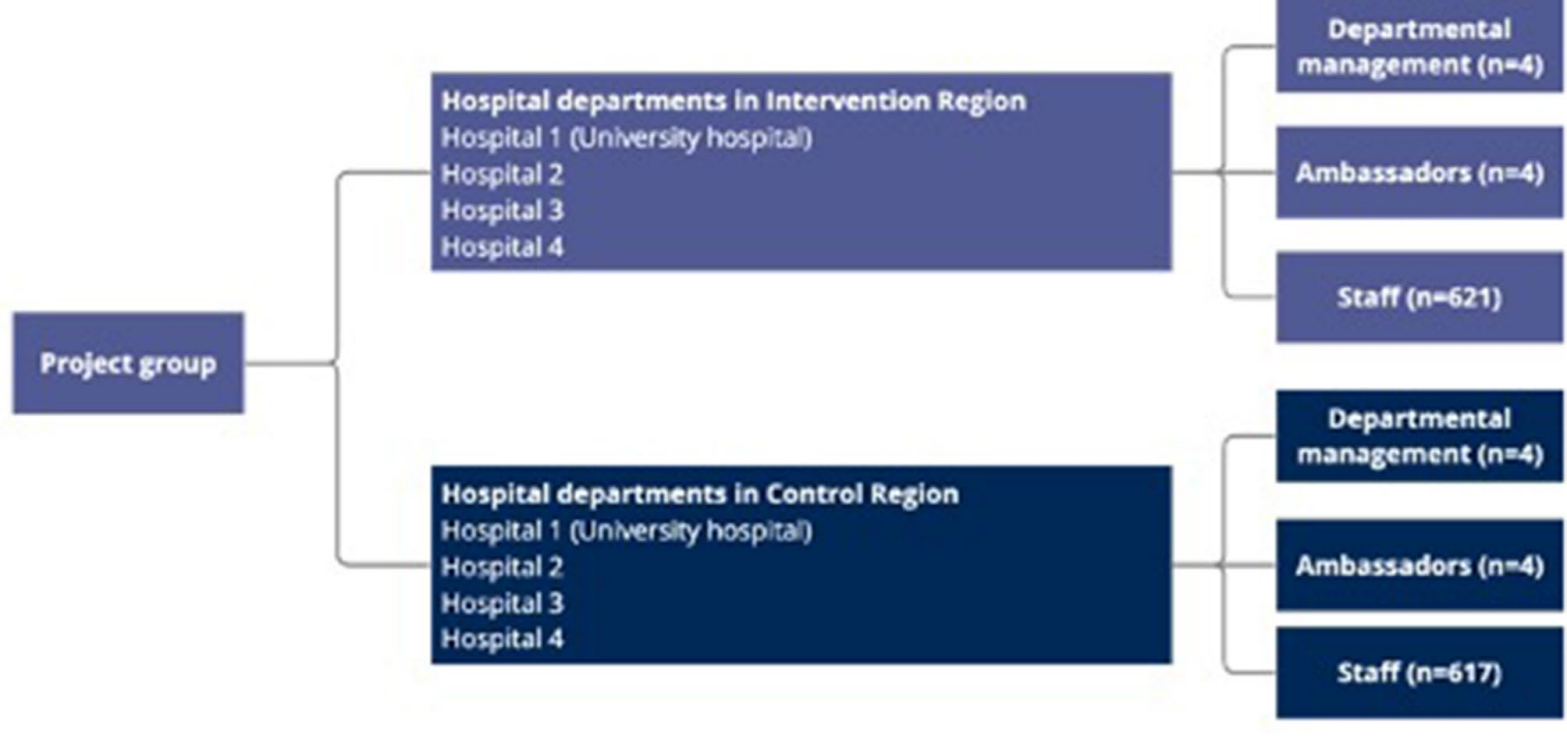
Overview of project group and collaborators

Management in all pediatric departments demonstrated their support by allocating time for simulation activities and granting permission for data collection. Additionally, each pediatric department was represented by a local ambassador who helped the management plan and conduct simulations, and ensure complete registration by simulation facilitators. A visual representation of the project group and collaborators can be found in Fig. 2.

#### Preparing simulation facilitators

Utilizing a ‘train the trainer’ methodology, simulation experts from a regional simulation center provide training and education to simulation facilitators. In the context of four pediatric departments, encompassing approximately 600 healthcare professionals, a group of 25 simulation facilitators had initially conducted simulation activities within their respective departments. To fulfill the demands for increased quantity and frequency of simulation-based team training, training additional simulation facilitators became imperative. In October 2022, 10 additional healthcare professionals from these pediatric departments were trained to become simulation facilitators. An intensive 3-day course effectively equipped these individuals, including doctors and nurses, with the skills necessary to proficiently lead simulation exercises within their departments. A detailed description of the simulation facilitator course can be seen in Appendix 3.

During March 2023, a cohort of 27 simulation facilitators from pediatric departments came together for a comprehensive 2-day workshop. This workshop was carefully crafted to support both newcomers and experienced simulation facilitators, with the overarching goal of fortifying a more robust and standardized foundation for the integration of simulation within their departments. The workshop delved into several critical areas, including acquainting participants with cutting-edge equipment, refining debriefing skills utilizing TeamGAINS [37], fostering a psychologically supportive learning atmosphere, and honing proficiency in executing simulation scenarios effectively. Further details and insights about the workshop can be found in Appendix 4.

Lastly, facilitators were provided with access to an online repository where they could access scenarios for conducting simulations. We expect these supportive initiatives to enable a higher number of performed simulations. Furthermore, we anticipate an enhanced quality of simulation in the intervention group.

#### Acquisition of equipment

To facilitate simulation activities in the pediatric departments in the intervention region, additional equipment was acquired. Equipment was selected based on pediatric mannequins, and pre-existing familiarity of facilitators in the region in terms of mannequin handling and preparation, as well as monitor and software user interfaces. Equipment will be placed on a rotation schedule, with each department having access to it for three weeks. After this period, a new rotation involving different equipment will commence. All equipment will undergo maintenance and servicing between rotation cycles. The project includes the following equipment:

- 2 x Premature Anne (premature).
- 2 x Sim NewB Light (newborn).
- 2 x Sim baby Light (9 months old).
- 2 x SimJunior Kid (6 years old).
- 4 x SimPads and 4 x monitors.

#### Registering all performed simulation

To achieve high-quality individual-level data on simulation training, local simulation ambassadors register all simulation training sessions in an online database, easily accessible from phone, tablet, or computer. Ambassadors are tasked with registering simulations in both the intervention group and the control group. This thorough registration process yields valuable information about all participants, including individual unique identification, profession, and name of their department. Furthermore, the specific date and time of the session and learning objectives in the scenarios are registered. This ongoing registration initiative spans from January 2023 to April 2024, which allows monitoring performed simulation three months before the start of the intervention. The registration process is supported by local ambassadors and performed to a similar degree in both intervention and control regions. Every three weeks, local ambassadors receive updates on the number of simulations registered within their respective departments. Ambassadors are responsible for ensuring the completeness of all registrations and conducting potential post-registrations.

## Feasibility

In 2018, members of the project group conducted a study using a train-the-trainer approach to establishing proof of concept and assessing feasibility. The study involved 53 healthcare professionals across various specialties employed at two Danish hospitals who participated in a simulation facilitator course. Following the course, all simulated scenarios were monitored over nine months. The feedback received from hospital management, simulation facilitators, and participants indicated that the simulation-based team training was perceived as relevant and beneficial [38]. Also, by conducting in situ simulations with healthcare professionals, improvements were observed in various aspects. These improvements included enhanced teamwork climate, safety climate, job satisfaction, perceptions of management, and working conditions [11, 12]. Lastly, we demonstrated a statistically significantly lower increase in sick leave over time among healthcare professionals participating in the simulation, when compared to a control group [17]. The findings highlight the positive impact of the simulation-based team training intervention on multiple dimensions, indicating its potential for fostering a collaborative and supportive healthcare environment. Further, the study demonstrated that a large-scale simulation-based team training intervention is feasible. However, the study included a relatively low exposure to simulation and lacked a control group in all but one of the studies [11, 12, 17].

## Evaluation

The evaluation process will include collecting data covering four separate outcome measurements. We hypothesize that the simulation-based team training program is related to teamwork climate and safety climate as well as job satisfaction. To test this hypothesis, we are investigating sick leave among staff (Study 1) and safety culture in pediatric departments (Study 2). Furthermore, we believe that simulation training including newborns is associated with clinical outcomes such as the Apgar score (Study 3). Lastly, we hypothesize that this simulation-based training intervention is related to cost-related benefits, prompting the need for a cost-benefit analysis (Study 4). Planned outcomes for evaluation are shown in Table 2, while a detailed plan for an analysis of each study is available in Appendix 5. Data in Studies 1, 2, and 3 are collected individually but will be represented by comparing larger groups to each other. The individual data though, will allow for a dose-response analysis, which will be conducted by examining outcomes to the actual extent of simulation-based team training performed, ranging from the highest to the lowest levels of training intensity.

### Analysis

Studies 1, 2, and 3 will each employ both paired and non-paired sample t-tests. The analysis will compare the groups (intervention group versus control group) as well as time periods (pre versus post). Thus, a difference-in-differences analysis will evaluate the intervention’s causal impact by comparing changes over time between groups. Normality assumptions will be evaluated using histograms to depict data distribution, whereas homogeneity of variance will be assessed via the SD test. Should any assumptions not hold, logarithm transformation or non-parametric tests (Wilcoxon) will be utilized.

**Table 2:** Measuring instruments for the effect evaluation

Outcome Measure	Objective	Instrument / data source	Variables	Time frame
**Study 1** Sick leave	To investigate if simulation-based team training is related to sick leave among healthcare professionals	BI^a^ Office, Central Denmark Region, and Documentation and Management Information, Region of Southern Denmark.	Main outcome: Rate of sick leave. Covariates: Gender, profession and age.	A) April 2022 to April 2023 (pre-intervention). B) April 2023 to April 2024 (during intervention) Anticipated: C) April 2024 to October 2024 (post-intervention).
**Study 2** Patient safety culture	To explore if the simulation intervention has an impact on patient safety culture	Electronic Safety Attitude Questionnaire Danish version (SAQ-DK)^b^ [38]. All participants completed a standard consent form in native language.	Main outcome: Patient safety culture dimensions including teamwork climate, safety climate, job satisfaction, stress recognition, perceptions of management, and working conditions. Covariates: Gender, profession, and years of experience.	A) April 2023 (pre-intervention). B) April 2024 (post-intervention).
**Study 3** Apgar score	To examine if simulation-based team training is associated with the treatment of critically ill newborns	BI^a^ Office, Central Denmark Region, and Documentation and Management Information, Region of Southern Denmark.	Main outcome: Difference in Apgar from one to 10 min after birth (Only newborns with an Apgar score less than 7 at one minute after birth are included).	A) April 2022 to April 2023 (pre-intervention) B) April 2023 to April 2024 (during intervention) Anticipated: C) April 2024 to October 2024 (post-intervention).
**Study 4** Cost benefit	To conduct a cost-benefit analysis, highlighting the potential return on investment associated to simulation-based team training	Not yet identified.	Costs associated with the intervention will be compared to potential financial benefits.	Post-intervention (Year 2025).

^a^ Business Intelligence

^b^ Safety Attitude Questionnaire - Denmark

### Sample size calculations

#### Study 1

Preliminary sick leave data from the BI Office, Central Denmark Region, has been utilized for the sample size calculation. The average rate of absence in the pediatric departments during the year 2022 was found to be 6.5%. This study does not include a superiority hypothesis, but rather a focus on exploring a potential relationship between simulation-based team training and sick leave. Thus, we did two analyses including a medium to small effect size, setting a Cohen’s d equal to 0.5 and another analysis setting the Cohen’s d equal to 0.25 [39]. Based on a Cohen’s d of 0.5, a sample size calculation using G*Power, states a necessary sample size of 210 individuals, whereas a Cohen’s d of 0.25 states a necessary sample size of 834 individuals. As we include a total of approximately 1,200 participants, we have included a sufficient number according to this sample size calculation. All parameters are illustrated in Appendix 5.

#### Study 2

Looking at a previous study, patient safety culture varied across dimensions at baseline, of which an average mean score was equal to 77.6% [11]. We do not plan to investigate a superiority hypothesis but rather focus on exploring a potential relationship between simulation-based team training and patient safety culture. As in study 1, we therefore did two analyses including two analyses assuming a medium to small effect size, setting a Cohen’s d equal to 0.5 and another analysis setting the Cohen’s d equal to 0.25 [39]. Based on a Cohen’s d of 0.5, a sample size calculation using G*Power, states a necessary sample size of 210 individuals, whereas a Cohen’s d of 0.25 states a necessary sample size of 834 individuals. By including a total of approximately 1,200 participants, we have comprised a sufficient number according to this sample size calculation. All parameters are illustrated in Appendix 5.

#### Study 3

Using the BI Office, Central Denmark Region, we have had access to the number of births at the four hospitals included in this project. During the year 2022, a total of 12,159 babies were born at these hospitals. Of these, we are only investigating newborns with an Apgar score of less than 7 one minute after birth. These newborns include 262 babies, leading to a total of approximately *n* = 524 across similar sizes of intervention and control groups. Once again, this study does not include a superiority hypothesis, but rather a focus on exploring a potential relationship between simulation-based team training and patient safety culture. As in studies 1 and 2, we therefore did two analyses including two analyses assuming a medium to small effect size, setting a Cohen’s d equal to 0.5 and another analysis setting the Cohen’s d equal to 0.25 [39]. Based on a Cohen’s d of 0.5, a sample size calculation using G*Power, states a necessary sample size of 210 individuals, whereas a Cohen’s d of 0.25 states a necessary sample size of 834 individuals. By including a total of approximately 524 newborns, we will only comprise a sufficient number in terms of the Cohen’s d equal to 0.5 though not the Cohen’s d equal to 0.25. All parameters are illustrated in Appendix 5.

#### Study 4

This study is in its preliminary stages, and we are yet to finalize the factors and variables to be collected. As a result, the specifics of statistical analysis have not been determined at this point.

### Expenses

Table 3 provides a detailed breakdown of all the project-related costs. To offer a thorough overview of the project’s expenditures, we present the following expenses:

#### Expense 1

Healthcare professionals performing a higher quantity of simulation.

#### Expense 2

Simulation facilitator course and workshop.

#### Expense 3

Equipment acquired from Laerdal including eight mannequins, four SimPads, four monitors, and a service contract.

#### Expense 4

Salaries associated with an assigned full-time PhD student and a supervisor employed at a 20% capacity within the project group.

**Table 3:** Overview of expenses

Expenditure	Elaborated
Healthcare professionals performing simulation	Nurses (*n* = 495)
	Resident doctor (*n* = 97)
	Specialist doctor (*n* = 98)
Simulation facilitator course and workshop	Facilitator course
	Workshop
Equipment	2 x Premature Anne
	2 x Sim NewB Light
	2 x Sim baby Light
	2 x SimJunior Kid
	4 x SimPad
	4 x Monitor
	1 x Service contract
Salary costs (over three years)	Full-time PhD student
	Supervisor employed at a 20%

## Discussion

Following the framework of the Medical Research Council, an intervention achieves complexity based on various factors inherent to the intervention [30]. These factors may include the study design, the range of behaviors targeted, the skills demanded from those delivering the intervention as well as the professions and experiences of those who receive the intervention [30]. We contend that the implemented simulation-based team training intervention meets the criteria for classification as a complex intervention. First, the intervention within this project revolves around simulation-based team training, which is being implemented across four departments during one year. Secondly, simulation-based team training targets various aspects of behaviors including among others teamwork, leadership, communication, and decision-making. Thirdly, there are certain skills required among simulation facilitators within this project. We conducted an instructor course and workshop intending to elevate the quality of simulation within the intervention group. Finally, the recipients of the intervention constitute a diverse group with varying levels of clinical experience as well as different professions, encompassing both doctors and nurses.

Following the framework outlined by the Medical Research Council framework for complex interventions, our description incorporates critical elements, including a feasibility study, development overview, implementation, and evaluation [30]. The data collection methods using register databases as well as online simulation registration, further contribute to the intricacy and depth of the intervention [40].

### Potential implications

Implementing this project over a one-year time frame makes it challenging to identify other factors potentially influencing the measured outcomes. In close collaboration with both departmental management and ambassadors, we are tracking possible unpredictable influencers. This could include the appointment of new management, cost savings within departments, and more. To our knowledge, no significant influencers threaten the intervention process as of now.

To achieve a noteworthy effect size within the project, it is crucial to ensure that the exposure to simulation-based team training is substantial. In comparison to the existing literature, our approach involves robust exposure, encompassing a year-long intervention with extensive participation in simulations [19–25].

A practical consideration pertains to the cooperation and engagement of stakeholders, as their support is pivotal for the intervention’s success. Collaboration with pediatric departments including management is essential, leading to their willingness to allocate time for simulation activities and grant permission for data collection. To ensure the greatest collaboration, we have proactively appointed local ambassadors from each department to ensure complete facilitator registration and foster strong partnerships with stakeholders throughout the study. Nonetheless, we cannot be entirely confident that the registration of conducted simulations is fully comprehensive. In the event of missing data, it is crucial to consider whether the absence of data is consistent across the intervention and control groups.

Another operational challenge revolves around the preparation and training of simulation facilitators. The efficacy of the intervention hinges on the competence and proficiency of these facilitators, who play a central role in guiding training sessions. To ensure their readiness, we have conducted comprehensive simulation facilitator courses and workshops, intending to standardize and enhance their abilities. Continuous support and communication with facilitators throughout the study period are vital to maintaining the intervention’s quality and consistency.

Moreover, the acquisition and maintenance of simulation equipment constitute practical considerations. The availability and proper functioning of equipment such as mannequins, SimPads, and monitors are imperative for successful simulations. In this endeavor, equipment has been shared among participating departments. Regular maintenance and coordination efforts are necessary to ensure the equipment remains in optimal condition throughout the study’s duration. Adequate resources and budget allocation for equipment upkeep and potential replacements are crucial to sustain the intervention’s effectiveness.

Although this study protocol thoroughly covers various practical and operational aspects, it’s essential to acknowledge some potential considerations. While simulation scenarios need to be tailored to the specific context, such as pediatrics, the concept of the online repository can be applied more broadly. Additionally, the clinical Apgar outcome is specific to pediatrics, while the other outcomes have a more generic nature and may have broader applicability.

## Conclusion

In conclusion, the successful execution of this trial hinges on addressing a range of practical and operational considerations that impact its feasibility and implementation. Collaboration with stakeholders, rigorous facilitator training, equipment management, participant engagement, and meticulous data collection are pivotal elements in this endeavor. By acknowledging these challenges and incorporating strategies to mitigate them, this study can effectively evaluate the impact of simulation-based team training on collaboration, communication, and patient outcomes within pediatric departments.

## Protocol amendments

In the event of important protocol modifications, our study has established a comprehensive plan for effective communication to ensure all relevant parties are informed promptly. This plan includes:

### Investigators

Any significant protocol modifications will be promptly communicated to all investigators involved in the study through official channels, such as email or in-person meetings, ensuring they are fully aware of the changes.

### Stakeholders

If necessary, local ambassadors and departmental management will be contacted directly to ensure effective communication and coordination at the local level.

### Ethical Committee

All protocol modifications requiring ethical committee approval will be submitted in a timely manner for their review and approval.

### Trial participants

Participants will be informed of any relevant changes to the study protocol that may affect their participation, rights, or safety. This will be done through appropriate channels, such as informed consent updates or direct communication.

Our commitment to transparent and effective communication ensures that all involved parties are kept informed of important protocol modifications, promoting study integrity and ethical conduct throughout the research process.

### Electronic supplementary material

Below is the link to the electronic supplementary material.

Supplementary Material 1

Supplementary Material 2

Supplementary Material 3

Supplementary Material 4

Supplementary Material 5

## Data Availability

Data concerning the study protocol appear in the main manuscript and in appendixes.

## Declarations

### Dissemination policy

We will regularly provide updates on ongoing results to participants and relevant stakeholders. Additionally, research findings will be disseminated through academic papers and presented at relevant conferences.
