# Supplementary material for: Simulation-based team training for healthcare professionals in pediatric departments: study protocol for a nonrandomized controlled trial
Source: BMC Med Educ. 2024 Jun 1;24:607. doi: 10.1186/s12909-024-05602-z (PMC11143636; doi:10.1186/s12909-024-05602-z)
Supplement: Supplementary file 1 — Supplementary Material 1 [file 12909_2024_5602_MOESM1_ESM.pdf]

Appendix 2: SPIRIT schematic diagram

		STUDY PERIOD			
	**Enrolment**	**Allocation**	**Post-allocation**		
**TIMEPOINT (months)**	**- 12 months**	**T0**	***T0***	***T1***	***T2***
**ENROLMENT:**					
**Eligibility screen**	X				
**Informed consent**	X				
Approval from hospital management	X				
**Allocation**		X			
**INTERVENTIONS:**					
Intervention			◆—	—	—◆
**ASSESSMENTS:**					
Registration of simulation			◆—	—	—◆
Sick leave among staff			X		X
Patient safety culture			X		X
Apgar score			X		X
Cost-benefit					X

*Recommended content can be displayed using various schematic formats. See SPIRIT 2013 Explanation and Elaboration for examples from protocols.
**List specific timepoints in this row.
