# Supplementary material for: Simulation-based team training for healthcare professionals in pediatric departments: study protocol for a nonrandomized controlled trial
Source: BMC Med Educ. 2024 Jun 1;24:607. doi: 10.1186/s12909-024-05602-z (PMC11143636; doi:10.1186/s12909-024-05602-z)
Supplement: Supplementary file 2 — Supplementary Material 2 [file 12909_2024_5602_MOESM2_ESM.pdf]

Appendix 3: Key elements in the simulation facilitator course for employees working within pediatrics.

Time period: On 10 to 12 October 2022

Key Elements to Report for Simulation-Based Research according to Cheng et al. 2016 [1]

**Elements**	**Sub-elements**	**Descriptor**	**Response**
Participant orientation	Orientation to the simulator	Describe how participants were oriented to the simulator (e.g., method, content, duration).	NA
	Orientation to the environment	Describe how participants were oriented to the environment (e.g., method, content, duration).	NA
Simulator type	Simulator make and model	Describe the simulator make and model.	NA
	Simulator functionality	Describe functionality and/or technical specifications that are relevant to the research question. Describe modifications, if any. Describe limitations of the simulator.	NA
Simulation environment	Location & Equipment	Describe where the simulation was conducted (e.g., in situ clinical environment, simulation center, etc.).	The simulation facilitator course took place at a simulation center
	Equipment	Describe the nature of the equipment available (e.g., type, amount, location, size, etc.).	Simulation specific equipment:<br>1 pcs.: Laerdal Premature Anne<br>1 pcs.: Laerdal Sim NewB Light<br>1 pcs.: Laerdal Sim baby Light<br>1 pcs.: Laerdal SimJunior Kid<br>3 pcs.: Medtronic LIFEPAK CR Plus, AED-trainers<br>1 pcs.: Physio Control Lifepak 15, defibrillator
	External stimuli	Describe any external stimuli (e.g., background noise).	NA
Simulation event/scenario	Event description	Describe if the event was programmed and/or scripted (e.g., orientation to event, scenario progression, triggers). If a scenario was used, the	Three scenarios were provided for the participants (Convulsions, CPR, anaphylaxis and sepsis) and the participants wrote and conducted four scenarios as well. The scenarios were used as a means for the

		scenario script should be provided as an appendix.	participant to put the presented theory into practice.
	Learning objectives	List the learning objectives and describe how they were incorporated into the event.	The following learning objectives were incorporated in the curriculum as oral presentations including; learning theory, crisis resource management, team psychological safety, the roles of a simulation instructor, use of standardized patients, briefing, scenario design, feedback, types of questions, and debriefing structure (TeamGAINS) [2].
	Group vs. individual practice	Describe if the simulation was conducted in groups or as individuals.	All simulations were conducted in groups of six. two functioned as simulation instructors facilitating the briefing, scenario and debriefing, and four as participants.
	Use of adjuncts	Describe if adjuncts (e.g., moulage, media, props) were used.	NA
	Facilitator/operator characteristics	Describe experience (e.g., clinical, educational), training (e.g., fellowship, courses), profession.	The educators in the simulation facilitator course were all experienced simulation instructors and were either trained nurses, physicians or Midwifes with a background in pediatrics, psychiatry, anesthesia or maternity.<br>The simulation operators were all medical students employed by the simulation center.
	Pilot testing	Describe if pilot testing was conducted (e.g., number, duration, frequency).	All scenarios included in course were tested by the course management prior to the intervention.
	Actors/confederates/standardized/simulated patients	Describe experience (e.g., clinical, educational), training (e.g., fellowship, courses), profession, sex. Describe various roles, including training, scripting, orientation, and compliance with roles.	The simulated patients (acting as parents) in this project were all medical students employed by the simulation center. They had all received previous training from an actor.
Instructional design (for educational interventions) or exposure (for simulation as investigative methodology)	Duration	Describe the duration of the educational intervention. If the intervention involves more than one segment, describe the duration of each segment.	The simulation facilitator course consisted of a three-day workshop using classroom teaching and practical performance of simulation to teach instructors basic skills in briefing, facilitating, and debriefing of simulation-based team training.
	Timing	Describe the timing of the educational intervention relative to the time when assessment/data collection occurs (e.g., just-in-time training).	The simulation facilitator course took place in October 2022.

	Frequency/repetitions	Describe how many repetitions were permitted and/or the frequency of training (e.g., deliberate practice).	All participants trained the role of facilitator twice during the course. The first time with a scenario provided by the course management and the second time with at scenario written by the participants.
	Clinical variation	Describe the variation in clinical context (e.g., multiple different patient scenarios).	NA
	Standards/assessment	Describe predefined standards for participant performance (e.g., mastery learning) and how these standards were established.	NA
	Adaptability of intervention	Describe how the training was responsive to individual learner needs (e.g., individualized learning).	NA
	Range of difficulty	Describe the variation in difficulty or complexity of the task.	NA
	Non-simulation interventions and adjuncts	Describe all other non-simulation interventions (e.g., lecture, small group discussion) or educational adjuncts (e.g., educational video), how they were used, and when they were used relative to the simulation intervention.	NA
	Integration	Describe how the intervention was integrated into curriculum.	NA
Feedback and/or debriefing	Source	Describe the source of feedback (e.g., computer, simulator, facilitator).	All feedback were provided by experienced simulation instructors.
	Duration	Describe the amount of time spent.	Approximately 30 minutes were spent on feedback after each simulation.
	Facilitator presence	Describe if a facilitator was present (yes/no), and if so, how many facilitators.	A facilitator was present for all simulations. One facilitator for every six participants.
	Facilitator characteristics	Describe experience (e.g., clinical, educational), training (e.g., fellowship, courses), profession, sex.	The Facilitators/educators were all experienced simulation instructors and were either trained nurses, physicians or Midwifes with a background in pediatrics, psychiatry, anesthesia or maternity.<br>The simulation operators were all medical students employed by the simulation center.
	Content	Describe content (e.g., teamwork, clinical, technical	Focus of the feedback were:<br>-The role as a new simulation instructor,

		skills, and/or inclusion of quantitative data, etc.).	-briefing,<br>-facilitating a scenario<br>-debriefing.
	Structure/method	Describe the method of debriefing/feedback and debriefing framework used (ie, phases).	Feedback on facilitated scenarios was conducted based on TeamGAINS [2].
	Timing	Describe when the feedback and/or debriefing was conducted relative to the simulation event (e.g., terminal vs. concurrent).	Feedback was provided immediately after the participants had completed their debriefing.
	Video	Describe if video was used (yes/no) and how it was used.	No
	Scripting	Describe if a script was used (yes/no) and provide script details as an appendix.	No

1. Cheng A, Kessler D, Mackinnon R, Chang TP, Nadkarni VM, Hunt EA, et al. Reporting guidelines for health care simulation research: extensions to the CONSORT and STROBE statements. Adv Simul [Internet]. Advances in Simulation; 2016;1:1–13. Available from: http://dx.doi.org/10.1186/s41077-016-0025-y
2. Kolbe M, Weiss M, Grote G, Knauth A, Dambach M, Spahn DR, et al. TeamGAINS: A tool for structured debriefings for simulation-based team trainings. BMJ Qual Saf. 2013;22:541–53.
