# Supplementary material for: Simulation-based team training for healthcare professionals in pediatric departments: study protocol for a nonrandomized controlled trial
Source: BMC Med Educ. 2024 Jun 1;24:607. doi: 10.1186/s12909-024-05602-z (PMC11143636; doi:10.1186/s12909-024-05602-z)
Supplement: Supplementary file 3 — Supplementary Material 3 [file 12909_2024_5602_MOESM3_ESM.pdf]

Appendix 4: Workshop program

Time period: March 30th and 31st 2023
Included individual workshops:

1) Introduction to new equipment
    - Laerdal equipment was demonstrated by two Laerdal employees
    - Equipment consisted of four mannequin types (premature Anne, Sim NewB Light, Sim Baby Light, and SimJunior Kid), simpads, monitors, and wifi-dongles
    - Attendees were able to work hands-on with the equipment
2) Team GAINS
    - The debriefing approach Team GAINS [1] is presented to attendees
    - The preferred debriefing method to be used by simulation facilitators while facilitating the simulation
3) Questioning methods
    - An advanced session on various types of questions the simulation facilitator can and should ask questions during the debriefing
4) The difficult debriefing
    - Advanced tools for how the simulation facilitator can deal with a difficult debriefing
5) Psychological safety and the safe learning space
    - A workshop that focused on how to apply psychological safety and furthermore to create a safe learning space, when facilitating simulation
6) Implementation of simulation in attendees' own department
    - A session that supports simulation facilitators in terms of implementing simulation in situ
7) Live simulation and feedback
    - A practical session in which attendees facilitated two simulation sessions including briefing, scenario, and debriefing
    - Four scenarios were facilitated in four workshop sessions
        - Scenario 1: Seizure (mannequin: Sim Baby Light / 14-month-old)
        - Scenario 2: Ventricular fibrillation (mannequin: SimJunior / 8-year-old)
        - Scenario 3: Resuscitation (mannequin: premature Anne / neonatal)
        - Scenario 4: Respiratory Syncytial Virus (mannequin: Sim NewB Light/newborn)
    - Participants received feedback from experienced simulation facilitators

References:

1. Kolbe M, Weiss M, Grote G, Knauth A, Dambach M, Spahn DR, et al. TeamGAINS: A tool for structured debriefings for simulation-based team trainings. BMJ Qual Saf. 2013;22:541–53.
