# Supplementary material for: Simulation-based team training for healthcare professionals in pediatric departments: study protocol for a nonrandomized controlled trial
Source: BMC Med Educ. 2024 Jun 1;24:607. doi: 10.1186/s12909-024-05602-z (PMC11143636; doi:10.1186/s12909-024-05602-z)
Supplement: Supplementary file 5 — Supplementary Material 5 [file 12909_2024_5602_MOESM5_ESM.pdf]

Appendix 5: Analysis plan

In terms of data management, the corresponding author (AS) will be responsible for overseeing all aspects related to coding, security, and storage. All statistical analyses will be performed using STATA software. A plan for analysis can be found on the following pages:

Study 1: Page 2-4
Study 2: Page 5-6
Study 3: Page 7-8
Study 4: Page 9
References: Page 10

Study 1

Title

Impact of simulation-based team training on sick leave among healthcare professionals: a multi-site nonrandomized controlled trial.

Objective

To investigate if simulation-based team training is related to sick leave among healthcare professionals.

Design and applied guidelines

Nonrandomized controlled trial using TREND guidelines [1].

Outcomes

Changes in the rate of sick leave among healthcare professionals.

Time frame

A) April 2022 to April 2023 (pre-intervention).

B) April 2023 to April 2024 (during intervention).

Anticipated: C) April 2024 to October 2024 (post-intervention).

Data source

BI (Business Intelligence) Office, Central Denmark Region[1], and Documentation and Management Information, Region of Southern Denmark[2].

Participants

N ≈ 1,200

Statistics/analysis

Purpose of analysis: We plan to compare the rate of sick leave across the intervention and control groups over time periods. The analyses will be conducted for all participants, as well as for healthcare professionals who have been employed in the same department during the whole study period, referred to as the 'complete-case analyses'. The rate of sick leave will be determined by dividing the hours of sick leave for each staff member by their corresponding portion of employed hours and subsequently multiplying the quotient by 100. Thus, part-time employment or change in the workplace will be taken into account. The rate of sick leave will be analyzed using the percentage rate of sick leave as well as the standard deviation.

Main analysis: To explore the objective concerning the relationship between simulation-based team training and sick leave, the main analysis includes a difference-in-difference analysis. Using an

[1] An ongoing administrative Human Resources database, covering all employment-related information in the Central Denmark Region

[2] An ongoing administrative Human Resources database, covering all employment-related information in the Region of Southern Denmark

unpaired t-test, this analysis will compare groups (intervention versus control group) over time (before versus after intervention).

Sub-analysis: Individual paired and unpaired t-tests will be applied to make comparisons across time periods and groups. We will add histograms illustrating the distribution of data. If data is not normally distributed, non-parametric tests (Wilcoxon signed-rank test and Mann–Whitney U rank sum test) will be applied.

Key assumption: In the absence of the intervention, the treatment and control groups would have followed parallel trends over time.

Power analysis/sample size calculations

t tests - Means: Difference between two independent means (two groups)
Analysis: A priori: Compute required sample size

**Input:**	Tail(s)	=	Two
	Effect size d	=	0.5
	α err prob	=	0.05
	Power (1-β err prob)	=	0.95
	Allocation ratio N2/N1	=	1
**Output:**	Noncentrality parameter δ	=	3.6228442
	Critical t	=	1.9714347
	Df	=	208
	Sample size group 1	=	105
	Sample size group 2	=	105
	Total sample size	=	210
	Actual power	=	0.9501287

t tests - Means: Difference between two independent means (two groups)
Analysis: A priori: Compute required sample size

**Input:**	Tail(s)	=	Two
	Effect size d	=	0.25
	α err prob	=	0.05
	Power (1-β err prob)	=	0.95
	Allocation ratio N2/N1	=	1
**Output:**	Noncentrality parameter δ	=	3.6098823
	Critical t	=	1.9628194
	Df	=	832
	Sample size group 1	=	417
	Sample size group 2	=	417
	Total sample size	=	834
	Actual power	=	0.9500922

Study 2

Title

Exploring the link between simulation-based team training and patient safety culture: a multi-site nonrandomized controlled trial.

Objective

To explore if simulation intervention is associated with patient safety culture.

Design and applied guidelines

Nonrandomized controlled trial using TREND guidelines [1].

Outcomes

Changes in patient safety culture among healthcare professionals.

Time frame

A) April 2023 (pre-intervention).

B) April 2024 (post-intervention).

Data source

Electronic Safety Attitude Questionnaire Danish version (SAQ-DK) [3].

Participants

N ≈ 1,200

Statistics/analysis

Purpose of analysis: We plan to compare the patient safety culture across the intervention and control groups over time periods. The analyses will be conducted for all participants, as well as for healthcare professionals who have been employed in the same department during the whole study period, referred to as the 'complete-case analysis'.

To calculate patient safety culture outcomes, every SAQ-DK item score will be converted to a 0–100 points scale in which 1=0, 2=25, 3=50, 4=75, and 5=100 [3]. To match positively worded questions, items number 2 and 11 are scored in reverse. Mean scale scores will be calculated using the average score of the scaled items.

Main analysis: To explore the objective in terms of whether a simulation intervention has an impact on patient safety culture, the main analysis includes a difference-in-difference analysis. Using an unpaired t-test, this analysis will compare groups (intervention versus control group) over time (before versus after intervention).

Sub-analysis: Individual paired and unpaired t-tests will be applied to make comparisons across time periods and groups. We will add histograms illustrating the distribution of data. If data is not normally distributed, non-parametric tests (Wilcoxon signed-rank test and Mann–Whitney U rank sum test) will be applied.

Key assumption: In the absence of the intervention, the treatment and control groups would have followed parallel trends over time.

Power analysis/sample size calculations

t tests - Means: Difference between two independent means (two groups)
Analysis: A priori: Compute required sample size

**Input:**	Tail(s)	=	Two
	Effect size d	=	0.5
	α err prob	=	0.05
	Power (1-β err prob)	=	0.95
	Allocation ratio N2/N1	=	1
**Output:**	Noncentrality parameter δ	=	3.6228442
	Critical t	=	1.9714347
	Df	=	208
	Sample size group 1	=	105
	Sample size group 2	=	105
	Total sample size	=	210
	Actual power	=	0.9501287

t tests - Means: Difference between two independent means (two groups)
Analysis: A priori: Compute required sample size

**Input:**	Tail(s)	=	Two
	Effect size d	=	0.25
	α err prob	=	0.05
	Power (1-β err prob)	=	0.95
	Allocation ratio N2/N1	=	1
**Output:**	Noncentrality parameter δ	=	3.6098823
	Critical t	=	1.9628194
	Df	=	832
	Sample size group 1	=	417
	Sample size group 2	=	417
	Total sample size	=	834
	Actual power	=	0.9500922

Study 3

Title

Investigating the influence of simulation-based team training on neonatal critical care: a multi-site nonrandomized controlled trial.

Objective

To examine if simulation-based team training is associated with the treatment of critically ill newborns.

Design and applied guidelines

Nonrandomized controlled trial using TREND guidelines [1].

Outcomes

Changes in low Apgar scores one minute after birth.

Time frame

A) April 2022 to April 2023 (pre-intervention).

B) April 2023 to April 2024 (during intervention).

Anticipated: C) April 2024 to October 2024 (post-intervention).

Data source

BI Office, Central Denmark Region, and Documentation and Management Information, Region of Southern Denmark.

Participants

N ≈ 524

Statistics/analysis

Purpose of analysis: We plan to compare Apgar scores among critically ill newborns across the intervention and control groups over time periods. Low Apgar scores at one minute after birth will be compared.

Main analysis: To explore if a simulation intervention influences the Apgar score, the main analysis includes a difference-in-difference analysis. Using an unpaired t-test, this analysis will compare newborns in groups (intervention versus control group) over time (before versus after intervention).

Sub-analysis: Individual paired and unpaired t-tests will be applied to make comparisons across time periods and groups. We will add histograms illustrating the distribution of data. If data is not normally distributed, non-parametric tests (Wilcoxon signed-rank test and Mann–Whitney U rank sum test) will be applied.

Key assumption: In the absence of the intervention, the treatment and control groups would have followed parallel trends over time.

Power analysis/sample size calculations

t tests - Means: Difference between two independent means (two groups)

Analysis: A priori: Compute required sample size

**Input:**	Tail(s)	=	Two
	Effect size d	=	0.5
	α err prob	=	0.05
	Power (1-β err prob)	=	0.95
	Allocation ratio N2/N1	=	1
**Output:**	Noncentrality parameter δ	=	3.6228442
	Critical t	=	1.9714347
	Df	=	208
	Sample size group 1	=	105
	Sample size group 2	=	105
	Total sample size	=	210
	Actual power	=	0.9501287

t tests - Means: Difference between two independent means (two groups)

Analysis: A priori: Compute required sample size

**Input:**	Tail(s)	=	Two
	Effect size d	=	0.25
	α err prob	=	0.05
	Power (1-β err prob)	=	0.95
	Allocation ratio N2/N1	=	1
**Output:**	Noncentrality parameter δ	=	3.6098823
	Critical t	=	1.9628194
	Df	=	832
	Sample size group 1	=	417
	Sample size group 2	=	417
	Total sample size	=	834
	Actual power	=	0.9500922

Study 4

Title

Return on investment for simulation-based team training: An evaluation of a cost-benefit analysis.

Objective

To conduct a cost-benefit analysis, highlighting the potential return on investment associated to simulation-based team training.

Design

An evaluation of a nonrandomized controlled trial according to the instructions of Recommendations for Conduct, Methodological Practices, and Reporting of Cost-effectiveness Analyses [5].

Outcomes

Costs saved versus costs used.

Time frame

Post-intervention (Year 2025).

Data source

BI Office, Central Denmark Region, and Documentation and Management Information, Region of Southern Denmark, as well as electronic patient records.

Participants

N/A.

Statistics/analysis

This study is in its preliminary stages, and we are yet to finalize the factors and variables to be collected. As a result, the specifics of statistical analysis have not been determined at this point.

References

1. Des Jarlais DC, Lyles C, Crepaz N. Improving the reporting quality of nonrandomized evaluations of behavioral and public health interventions: the TREND statement. Am J Public Health. 2004;94:361–6.

2. Lakens D. Calculating and reporting effect sizes to facilitate cumulative science: a practical primer for t-tests and ANOVAs. Front Psychol. 2013;4:863.

3. Kristensen S, Sabroe S, Bartels P, Mainz J, Christensen KB. Recommendations for Conduct, Methodological Practices, and Reporting of Cost-effectiveness Ana. Clin Epidemiol. 2015;7:149–60.

4. Schram A, Paltved C, Lindhard M, Kjaergaard-Anders G, Jensen H, Kristensen S. Patient safety culture improvements depend on basic healthcare education: A longitudinal simulation-based intervention study at two Danish hospitals. BMJ Open Qual. 2022.

5. Sanders GD, Neumann PJ, Basu A, Brock DW, Feeny D, Krahn M, et al. Recommendations for Conduct, Methodological Practices, and Reporting of Cost-effectiveness Analyses: Second Panel on Cost-Effectiveness in Health and Medicine. JAMA. 2016;316:1093–103.
